# Bartter Syndrome With Recurrent Hypokalemic Periodic Paralysis: A Case Report

**DOI:** 10.7759/cureus.72406

**Published:** 2024-10-25

**Authors:** Arki Das, Rohini R, Somnath Panda

**Affiliations:** 1 Internal Medicine, All India Institute of Medical Sciences, Raipur, Raipur, IND; 2 Medicine, All India Institute of Medical Sciences, Raipur, Raipur, IND

**Keywords:** acute flaccid paralysis, bartter syndrome, genetic mutations, growth retardation, hypocalcemia, hypokalemia, metabolic alkalosis, renal tubular disorder

## Abstract

Hypokalemia is known to manifest as neurological weakness and cardiac rhythm disturbances. Severe hypokalemia can be life-threatening and needs prompt recognition and management. However, the workup for hypokalemia is equally essential to prevent future recurrences and complications. Bartter syndrome is one of the rare causes of hypokalemia, usually presenting early in childhood with growth retardation and failure to thrive. It is rare for a case to present in late adolescence. Here, we report a case who presented with recurrent hypokalemic paralysis and short stature in adolescence. We highlight the approach to hypokalemia and emphasize the need for early diagnosis to prevent potential complications.

## Introduction

In 1962, Bartter et al. identified a new syndrome characterized by hypokalemia and metabolic alkalosis, hyperaldosteronism, and hyperplasia of the juxtaglomerular apparatus (JGA) with normal blood pressure, primarily affecting young individuals [1]. Bartter syndrome has an estimated prevalence of one in 1,000,000 individuals [2]. It refers to a group of renal diseases caused by defective salt reabsorption in the thick ascending limb (TAL) of the loop of Henle, resulting in salt wasting and electrolyte imbalances such as hypokalemia, hypercalciuria, and hypochloremic metabolic alkalosis [3]. One of the serious manifestations of Bartter syndrome is recurrent hypokalemic periodic paralysis, where episodes of muscle weakness or paralysis occur due to critically low potassium levels [4]. Adult-onset classic Bartter syndrome is rare, with only a few cases reported to date. We present a case of adult-onset Bartter syndrome presenting with acute flaccid paralysis.

## Case presentation

History

A 15-year-old male patient presented to the emergency department with complaints of loose stools and vomiting, followed by weakness in all four limbs for one day. The weakness had a rapid onset, causing difficulty walking, sitting up from a lying position, and putting on slippers. There was no history of head drop or bladder or bowel incontinence. There was no associated tingling or numbness in any of the limbs. There was no difficulty in swallowing food. There was no history suggestive of antecedent viral illness, vaccination, or any animal bite. A similar episode of weakness in all four limbs occurred one year ago, lasting for three to four days, and subsided with medication. This prior episode was also preceded by gastrointestinal symptoms, including two episodes of loose stools and approximately 10-12 episodes of non-bilious, non-projectile vomiting.

Clinical examination

The patient was conscious and oriented to time, place, and person, with a Glasgow Coma Scale (GCS) score of 15/15. He was of short stature (height: 128 cm, weight: 28 kg). Vital signs included blood pressure (BP) of 100/72 mmHg, heart rate (HR) of 78 bpm, respiratory rate (RR) of 18/minute, and 98% SpO2 on room air. There were no signs of pallor, icterus, cyanosis, clubbing, or edema.

Neurological examination revealed normal higher mental functions and symmetrical muscle bulk. Cranial nerve findings were normal. In all limbs, tone and power were decreased, with the following power ratings as shown in Table 1. Cardiovascular, respiratory, and abdominal findings were unremarkable.

**Table 1:** Motor strength assessment of limbs

Movement	Right	Left
Shoulder flexion/extension	3/5	3/5
Elbow flexion/extension	3/5	3/5
Hip flexion	2/5	2/5
Hip extension	2/5	2/5
Hip abduction	2/5	2/5
Hip adduction	2/5	2/5
Knee flexion	3/5	3/5
Knee extension	3/5	3/5
Ankle dorsiflexion	4/5	4/5
Ankle plantarflexion	3/5	3/5

Investigations and management

**Figure 1:**
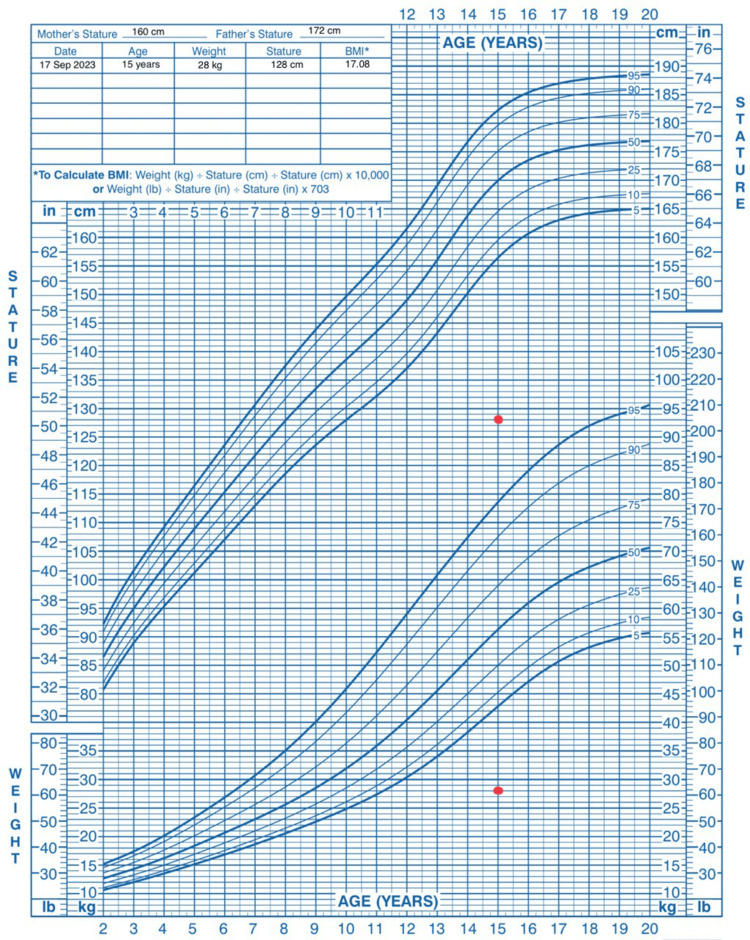
Anthropometric growth chart of the patient compared to CDC growth percentiles This growth chart is reproduced from the CDC Growth Charts (United States), with modifications to indicate the patient's stature and weight. The patient's stature and weight are indicated by red dots on the chart [5]. CDC: Centers for Disease Control and Prevention, BMI: body mass index

Initially, complete blood count (CBC), renal function tests (RFTs), arterial blood gas (ABG), liver function tests (LFTs), and other serum electrolyte tests were advised. ABG revealed hypokalemic metabolic alkalosis. Serum electrolytes revealed hypokalemia, hypomagnesemia, and hypocalcemia. Urine spot potassium was high, with a transtubular potassium gradient of 6, suggesting renal loss, specifically from the distal tubule. The calcium/creatinine (Ca/Cr) ratio was high, indicating Bartter syndrome. The ultrasound (USG) of the abdomen was unremarkable. Growth charts (Figure 1) indicated stunted growth (<5th percentile for stature-for-age and weight-for-age). The patient experienced two episodes of tetany in-hospital, which were promptly managed with IV calcium gluconate and an injection magnesium. In addition, the patient received IV and oral potassium chloride and spironolactone. Serum potassium values gradually improved, and the patient's motor power showed improvement. Genetic testing for Bartter syndrome was discussed but deferred due to financial constraints.

## Discussion

Bartter syndrome is an inherited rare congenital salt-wasting renal tubular disease characterized by hyperaldosteronism with hypokalemic hypochloremic metabolic alkalosis with normal blood pressure [6]. Different variants of Bartter syndrome arise from different gene mutations that affect the transporters responsible for salt reabsorption in the TAL [7]. Findings that suggest hypokalemic metabolic alkalosis in children and young patients should prompt suspicion of Bartter and Gitelman syndrome, both of which present similarly and can be distinguished with features as shown in Table 2 [7].

**Table 2:** Differentiating features between Bartter and Gitelman syndrome Data source: [7] *SLC12A1*: solute carrier family 12 member 1, *KCNJ1*: potassium voltage-gated channel subfamily J member 1, *CLCNKB*: chloride voltage-gated channel Kb, *BSND*: barttin CLCNK-type chloride channel accessory beta subunit, *SLC12A3*: solute carrier family 12 member 3

Feature	Bartter syndrome	Gitelman syndrome
Genetic mutations	*SLC12A1*,* KCNJ1*, *CLCNKB*, *BSND*	*SLC12A3*, *CLCNKB*
Age of presentation	Infancy or early childhood	Late childhood or adulthood
Symptoms	Polyuria, polydipsia, growth retardation, nephrocalcinosis (sometimes)	Muscle cramps, weakness, fatigue, tetany
Evidence of dehydration	Often present	Often absent
Serum magnesium	Typically normal	Decreased
Urinary calcium	Normal/increased	Decreased
Maternal hydramnios	Common (neonatal type), rare (classical type)	Absent
Urinary prostaglandins	High	Normal
Response to prostaglandins inhibitors	Good	Rare
Prognosis	Variable, potential for progressive renal damage	Generally a better prognosis

Types of Bartter syndrome

Bartter syndrome is classified into five types based on the genes involved, as shown in Table 3. Confirmation can be achieved by genetic testing, such as next-generation sequencing, for the genes solute carrier family 12 member 1 (*SLC12A1*), potassium voltage-gated channel subfamily J member 1 (*KCNJ1*), chloride voltage-gated channel Kb (*CLCNKB*), chloride voltage-gated channel Ka (*CLCNKA*), barttin CLCNK-type chloride channel accessory beta subunit (*BSND*), and melanoma antigen family D2 (*MAGED2*) [2,7-9].

**Table 3:** Different clinical features associated with different Bartter syndrome subtypes Data sources: [7-9] *SLC12A1*: solute carrier family 12 member 1, *KCNJ1*: potassium voltage-gated channel subfamily J member 1, *CLCNKB*: chloride voltage-gated channel Kb, *BSND*: barttin CLCNK-type chloride channel accessory beta subunit, *CLCNKA*: chloride voltage-gated channel Ka, *MAGED2*: melanoma antigen family D2, ClC-Kb: chloride channel Kb, ClC-Ka: chloride channel Ka

Type of Bartter syndrome	Gene	Inheritance	Protein	Clinical findings
Type 1	SLC12A1	Autosomal recessive	Na-K-2Cl co-transporter (NKCC2) in the thick ascending limb of the loop of Henle	Severe prenatal polyhydramnios, preterm birth, failure to thrive in infancy, hypokalemic, metabolic alkalosis, hypercalciuria, nephrocalcinosis, hyposthenuria, salt wasting, do not survive till adulthood
Type 2	KCNJ1	Autosomal recessive	Potassium inward rectifying channel (ROMK) in the thick ascending limb of the loop of Henle	Similar to type 1, including prenatal polyhydramnios, preterm birth, failure to thrive, hypokalemic metabolic alkalosis, hypercalciuria, nephrocalcinosis, muscle weakness, do not survive till adulthood
Type 3 (classical)	CLCNKB	Autosomal recessive	ClC-Kb in the thick ascending limb of the loop of Henle	Milder form compared to type 1 and 2, normal to mildly reduced prenatal fluid levels, hypokalemic hypochloremic metabolic alkalosis, normal to mild hypercalciuria (may or may not develop nephrocalcinosis), muscle cramps, fatigue, growth retardation
Type 4a	BSND	Autosomal recessive	Barttin (regulates ClC-Ka and ClC-Kb chloride channels in the kidneys and inner ear)	Severe prenatal polyhydramnios, preterm birth, hypokalemia, hypochloremic metabolic alkalosis, hypercalciuria, nephrocalcinosis, sensorineural deafness, does not survive till adulthood
Type 4b	*CLCNKA* and *CLCNKB* (both genes affected)	Autosomal recessive	ClC-Ka and ClC-Kb	Similar to type 4a, with severe prenatal polyhydramnios, preterm birth, sensorineural deafness, hypokalemia, metabolic alkalosis, nephrocalcinosis, failure to thrive, do not survive till adulthood
Type 5	MAGED2	X-linked recessive	MAGED2	Polyhydramnios (resolves after birth), hypokalemia, metabolic alkalosis, hypomagnesemia, transient antenatal Bartter-like syndrome that resolves with age, no nephrocalcinosis

**Figure 2:**
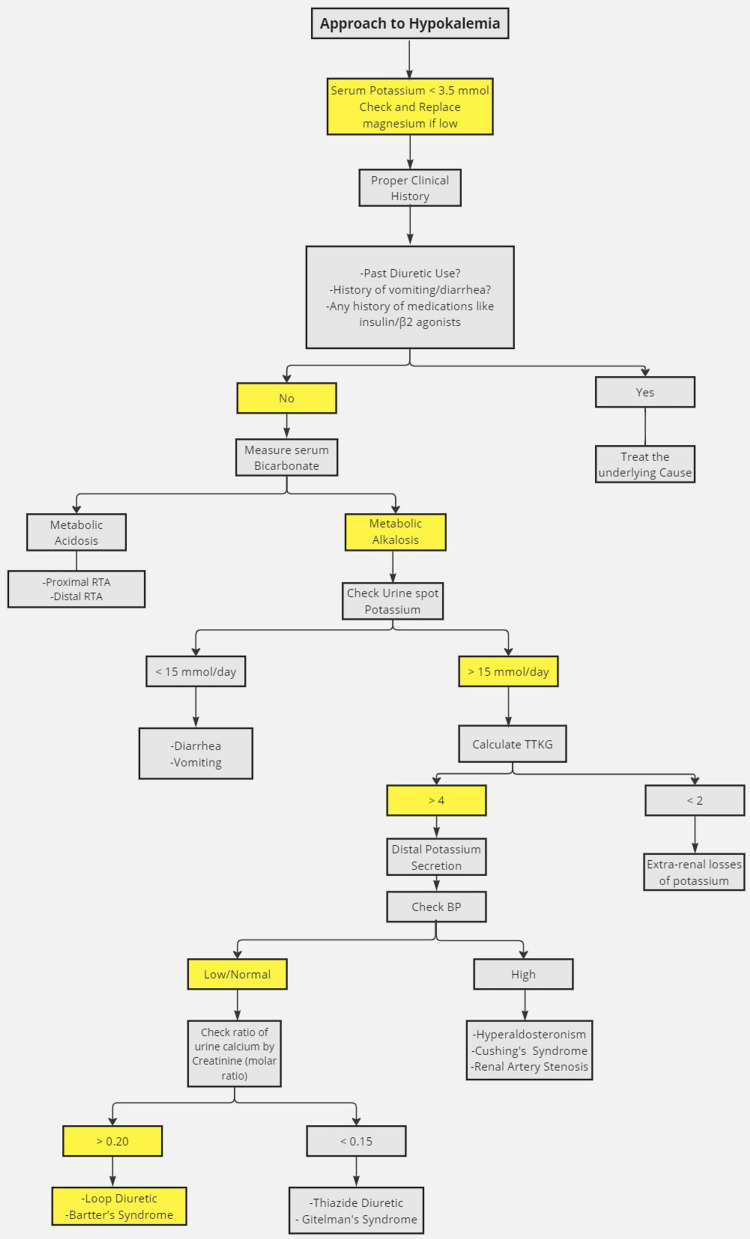
Approach to hypokalemia Highlighted sections in the flowchart specifically illustrate this case's findings. Recreated with modifications from Aminy et al. [10] and Cao et al. [11] RTA: renal tubular acidosis, TTKG: transtubular potassium gradient, BP: blood pressure, pH: potential of hydrogen, mmol/L: millimoles per liter, mmol/day: millimoles per day

A thorough workup is needed to find the etiology of hypokalemia. The investigations include transtubular potassium gradient (TTKG), urinary potassium, urinary chloride, urinary calcium/creatinine ratio, and blood pressure monitoring. Figure 2 illustrates a stepwise approach to ordering these investigations and interpreting the results [10,11].

Pseudohypokalemia is defined by a difference of over 0.4 mmol/L between serum and plasma potassium levels when samples are kept at room temperature and tested within one hour of collection [12].

Transcellular shifts refer to the abrupt movement of potassium from extracellular to intracellular fluid in cells. Examples include insulin excess, hyperthyroidism, beta-2 adrenergic agonists, and chloroquine [13].

Our patient's clinical and biochemical profile, including growth retardation and electrolyte imbalances, suggested type 3 Bartter syndrome. Management typically involves potassium chloride supplementation, prostaglandin inhibitors such as indomethacin, and aldosterone antagonists such as spironolactone [12-14]. Angiotensin-converting enzyme inhibitors may be prescribed to address proteinuria or to assist in correcting low potassium levels [15,16]. Additionally, maternal treatment with nonsteroidal anti-inflammatory drugs (NSAIDs) during antenatal presentations provides benefits [3]. Reports have indicated the onset of focal segmental glomerulosclerosis (FSGS) in Bartter syndrome due to the continued activation of the renin-angiotensin system, leading to secondary chronic glomerular hyperfiltration [17].

Our patient was a young man with constitutional growth retardation, delayed puberty, mild hydronephrosis, hypokalemia, hyponatremia, hypomagnesemia, normal blood pressure, hypochloremic metabolic alkalosis, and raised urinary Ca/Cr ratio suggesting Bartter type 3 (classical Bartter) syndrome. Loose stools followed each episode of hypokalemic paralysis, and the treatment involved intravenous potassium chloride. We administered a trial of growth hormone supplementation to help the patient catch up on growth during adolescence. Although the parents received counseling about genetic testing, they decided to defer it due to financial constraints.

## Conclusions

Although rare, Bartter syndrome should be in the differential diagnosis of adults with acute flaccid paralysis, hypokalemia, and metabolic alkalosis. This case illustrates, as others have, the diagnostic and management difficulties of Bartter syndrome, notably when recurrent episodes of hypokalemic periodic paralysis are a prominent feature. In that regard, a structured algorithm on the approach to hypokalemia would be critical in ensuring systemic evaluation for the timely diagnosis of underlying conditions such as Bartter syndrome. Early interventions would prevent complications such as growth retardation and improve outcomes in young patients.
